# Evaluating Artificial Intelligence in Clinical Settings—Let Us Not Reinvent the Wheel

**DOI:** 10.2196/46407

**Published:** 2024-08-07

**Authors:** Kathrin Cresswell, Nicolette de Keizer, Farah Magrabi, Robin Williams, Michael Rigby, Mirela Prgomet, Polina Kukhareva, Zoie Shui-Yee Wong, Philip Scott, Catherine K Craven, Andrew Georgiou, Stephanie Medlock, Jytte Brender McNair, Elske Ammenwerth

**Affiliations:** 1 Usher Institute The University of Edinburgh Usher Building Edinburgh United Kingdom; 2 Amsterdam UMC University of Amsterdam Medical Informatics Amsterdam Netherlands; 3 Amsterdam Public Health Research Institute, Digital Health and Quality of Care Amsterdam Netherlands; 4 Australian Institute of Health Innovation Macquarie University Sydney Australia; 5 Institute for the Study of Science, Technology and Innovation The University of Edinburgh Edinburgh United Kingdom; 6 School of Social, Political and Global Studies and School of Primary, Community and Social Care Keele University Keele United Kingdom; 7 Faculty of Medicine, Health and Human Sciences Macquarie University Sydney Australia; 8 Department of Biomedical Informatics University of Utah Utah, UT United States; 9 St. Luke’s International University Tokyo Japan; 10 University of Wales Trinity St David Swansea United Kingdom; 11 University of Texas Health Science Center San Antonio, TX United States; 12 Amsterdam Public Health, Methodology & Aging & Later Life Amsterdam Netherlands; 13 Department of Health Science and Technology Aalborg University Aalborg Denmark; 14 Institute of Medical Informatics Private University for Health Sciences and Health Technology UMIT TIROL Hall in Tirol Austria

**Keywords:** artificial intelligence, evaluation, theory, patient safety, optimisation, health care, optimization

## Abstract

Given the requirement to minimize the risks and maximize the benefits of technology applications in health care provision, there is an urgent need to incorporate theory-informed health IT (HIT) evaluation frameworks into existing and emerging guidelines for the evaluation of artificial intelligence (AI). Such frameworks can help developers, implementers, and strategic decision makers to build on experience and the existing empirical evidence base. We provide a pragmatic conceptual overview of selected concrete examples of how existing theory-informed HIT evaluation frameworks may be used to inform the safe development and implementation of AI in health care settings. The list is not exhaustive and is intended to illustrate applications in line with various stakeholder requirements. Existing HIT evaluation frameworks can help to inform AI-based development and implementation by supporting developers and strategic decision makers in considering relevant technology, user, and organizational dimensions. This can facilitate the design of technologies, their implementation in user and organizational settings, and the sustainability and scalability of technologies.

## Introduction

The last two decades have seen rapid growth in artificial intelligence (AI) initiatives in health care settings, driven by the promises of improved treatment, quality, safety, and efficiency [1]. AI systems are computer algorithms that are able to mimic human intelligence to perform tasks. They are potentially capable of improving clinical decision-making. However, there is currently a lack of high-quality evidence of effectiveness, and an overoptimism regarding AI-based technologies in health care [2,3]. Many existing algorithms and applications fail to scale and migrate across settings [4], potentially leading to missed benefits or compromised patient safety.

Evidence from other sectors, such as finance and retail, may have limited applicability given the particular social, economic, technical processes, and legal challenges of health and social care settings [5]. Across the digital economy, AI has been successfully applied to historical data, for example, in financial forecasting [6] or retail marketing, where personalized advertisements have transformed consumer behavior [7]. These methods are harder to deploy in the more complex and sensitive settings of health and social care [5]. This is largely because developers and implementers focus on tool development and do not sufficiently draw on existing work to inform the conception and design of technologies, their use and optimization, and organizational strategies to implement them.

Theory-informed approaches to evaluation can help to ensure that technologies are effectively validated, implemented, and adopted. They can also help to ensure that systems do not result in unintended negative consequences, such as inappropriate or suboptimal care, exacerbated inequities, or clinician burnout [8]. Theories seek to explain complex relationships at an abstract level and can help to integrate a particular implementation with the empirical evidence base. As such, theory-informed evaluation frameworks can enable learning from experience, thus guiding developers, implementers, and evaluators through development, implementation, and optimization [9]. Ideally, the real-world experience gathered during this process is then used also to inform the refinement of evaluation frameworks.

Despite significant investments, there are currently only a few examples of the use of AI-based systems in health care and most systems are only beginning to be rolled out and embedded [10-12]. This is in contrast to the finance and retail sectors, where processes and products are standardized. To date, most activity has focused on diagnostic image-based systems and text or language processing, while complex precision medicine efforts are in very early stages of development. We here call for the increasing use of theory-informed approaches to evaluation to help ensure that developed systems can be adopted, scaled, and sustained within settings of use, and are safe and effective. Until now, this has not been done consistently, which has resulted in limited learning and limited ability to transfer learning across settings, as well as limited clinical and patient reassurance. If done appropriately, the implications for clinical settings are significant, as validated new knowledge can be disseminated and shared. This, in turn, obviates the need to learn through experience that can be painful, dangerous, and costly.

Unfortunately, despite increasing attention in research, the current application of theory-informed strategy and evaluation in AI practice is relatively limited in both health care and other sectors [13]. This may be due to a lack of understanding surrounding the theoretical literature (ie, why theories are useful in practice and how they may be used by different stakeholders), and the immediate focus of developers on demonstrating that technology works. Politically and managerially, there may be a drive to show modernization processes rather than making clinical and organizational decisions based on evidence-based outcomes. Where theories have been applied, these have been driven by business approaches to value creation in organizations [14], or by approaches designed to influence consumer behavior [15]. In these contexts, they have been strategically used to help address a particular stakeholder need (eg, how to maximize value through implementing AI in organizations and how to get consumers to accept AI technology). In health care, the range of stakeholders and associated needs however varies significantly from other sectors. While the managers and policymakers may focus on value and efficiency, patients are likely to be concerned about avoidable illness, and practitioners may focus on workloads and potential liability.

It is therefore often difficult to know what needs (and consequently what theory) to focus on and in what context. For example, while developers of technology now increasingly draw on cocreation with users to promote the adoption of AI, these approaches may not consider organizational drivers, workflow integration, multiplicity of stakeholders, or ethical considerations in implementation, thereby limiting the scalability of emerging applications.

Theory-informed approaches to evaluation in health care must be considered within their specific context, recognizing their relative positions and identifying which needs they address at various stages of the technology lifecycle. We aim to begin this journey by providing a conceptual overview of existing theory-informed frameworks that could usefully inform the development and implementation of AI-based technologies in health care. Despite some differences in technological properties and performance between AI- and non–AI-based technologies (Table 1) [16], many existing frameworks are likely to be applicable.

**Table 1:** Differences between artificial intelligence (AI)–based and non–AI-based health IT.

Applications	AI-based	Evidence	Non–AI-based	Evidence
Health services management	AI can help in optimizing resource allocation, scheduling, and workflow management by analyzing large data sets and identifying patterns and trends. For example, modeling of waiting times and underlying reasons	Limited evidence in relation to impact, mainly in relation to proof-of-concept [17,18]	Non–AI-based approaches typically rely on manual processes and human decision-making for resource management, scheduling, and workflow optimization. For example, patient flow management applications	High potential of data-driven approaches to improve organizational performance [19,20]
Predictive medicine	AI algorithms can analyze patient data, genetic information, and medical records to predict disease risks, treatment outcomes, and responses to therapies. This enables personalized medicine and targeted interventions	Many proof-of-concept studies but limited evidence in relation to how outputs are incorporated into clinical decision-making [21,22]	Non–AI-based approaches rely on statistical analysis and clinical expertise to make predictions about disease risks, treatment outcomes, and responses to therapies	Many proof-of-concept studies but limited evidence in relation to how outputs are incorporated into clinical decision-making [23,24]
Clinical decision support systems	AI to analyze large amounts of medical literature, patient data, and clinical guidelines to support clinical decision-making	Area of most focus, especially in imaging applications, AI has the potential to improve practitioner performance [25,26], but limited evidence surrounding organizational impacts or patient outcomes	Non–AI-based approaches rely on the expertise and experience of health care professionals, along with clinical guidelines and published research, to make clinical decisions	Demonstrated benefits for practitioner performance and patient outcomes in some areas of use (eg, drug-drug interactions) [27,28]
Laboratory and radiology information systems	Use of AI to detect abnormalities and to enhance the accuracy of diagnoses	Most progress has been made in relation to imaging [29,30], but limited attention has been paid to integration with organizational practices as above [26]	Non–AI-based diagnostics typically rely on visual inspection by health care professionals and manual analysis of patient data	This is associated with information overload but does take account of contextual factors
Patient data repositories	AI algorithms can process patient data to identify trends, patterns, and risk factors	Promising proof-of-concept studies, but limited implementation [31,32]	Patient data are stored in a centralized repository	Some evidence that digitized records and repositories can lead to improved quality, safety, and efficiency, but hard to assess and take a long time to materialize [33,34]
Population health management	Precision prevention approaches to identify populations at risk and tailor preventative interventions	Promising approaches to precision prevention in specific cohorts, but limited implementation [35,36]	Understanding factors that influence health outcomes and developing tailored interventions	Significant evidence of population health interventions [37,38]
Patient portals	AI-based symptom checkers and triage tools	Inconsistent evidence in relation to symptom checkers and triage tools, concerns in relation to diagnostic accuracy [39,40]	Access to generic informational resources	Tailored informational resources can improve satisfaction, involvement, and decision-making [41,42]
Telehealth and telecare	Online health assistants and chatbots	Mixed evidence of effectiveness usability and user satisfaction [43,44]	Access to generic informational resources.	Tailored informational resources can improve satisfaction, involvement, and decision-making [41,42]
Health information exchange	Extracting and converting unstructured or semistructured data into a standardized format	The use of free text data is still in its infancy but is promising. There is limited data on integration with existing ways of working and organizational functioning. [45,46]	Coding and transfer into standardized formats are often done by health care staff	Increased workloads for health care staff and coding are often not done accurately [47,48]

We here provide a conceptual overview of existing frameworks, focusing on practical applications of examples of existing theory-informed frameworks and their potential application to AI-based technologies in health care [49]. Frameworks were selected as examples illustrating these extracted categories. This work is not intended to be exhaustive but to provide a pragmatic introduction to the topic for nonspecialists [50,51].

To categorize frameworks in a meaningful way, we focused on their potential area of application and the particular interest or focus of various stakeholder groups who may need to draw on existing experience to inform their current efforts to develop, implement, and optimize AI-based technologies in health care settings.

## Health IT Evaluation Frameworks and Their Potential Application to AI

### Overview

The 3 distinct dimensions identified are illustrated in Table 2, along with potential applications of AI-based technologies in health care and example use cases. These include frameworks with a technology, user, and organizational focus. We discuss each of these categories, the application of exemplary frameworks, and practical implications for various stakeholders in the paragraphs below.

However, it is important to recognize that the categorization of frameworks provided here is a simplification. Various frameworks have common and, in some instances, overlapping elements. The categories presented are intended to facilitate navigation and application.

**Table 2:** Examples of the focus of existing health IT evaluation frameworks and their potential application to artificial intelligence.

Focus of the framework	Area of application	Example theoretical lenses	Practical implications	Stakeholders	Examples
Technology focus	Informing the conception and design of technologies To help AI^a^ system developers design a system that is usable and useful within intended use settings	Human-centered design	Actively and iteratively involve end users in system design and development	End users and developers	A team had developed an algorithm to predict arterial fibrillation from electrocardiograms, but prospective users stated that the information would not change their practice [10]
User focus	Informing and helping to optimize the use of technologies To help developers and implementers understand the various contexts of use of AI as well as unintended consequences, and tailor systems to maximize benefits and minimize harms	Sociotechnical systems	Plan with users to effectively integrate the system in their work practices and monitor progress over time	End users, implementers	IBM Watson encountered adoption-related issues, including usability and perceived usefulness of their oncology software, which eventually led to the abandonment The system increased the workloads of doctors and made treatment recommendations that were viewed as unsafe by doctors [52]
Organizational focus	Informing organizational strategies to implement technologies To help AI system implementers integrate AI safely within existing organizational structures and processes	Institutional theory	Plan and monitor how systems and their outputs are integrated within and across organizational units and existing technological and social structures	End users, organizational stakeholders, and implementers	Babylon Health UK (an AI-based remote service provider) failed because it did not fit with existing health system financing structures and cultures Many patients from outside the local area enrolled in the service, which meant that the product was not commercially viable for local organizations [53]

^a^AI: artificial intelligence.

### Frameworks With a Technology Focus

Many current AI applications in health care settings have been developed by AI specialists in laboratory settings. Consequently, they have struggled to successfully translate into clinical settings and deliver the performance achieved in research trials [54]. Frameworks with a technology focus can help to inform the “conception and design” of technologies, thereby helping to ensure that AI system developers design a system that is readily implemented and useful within intended use settings. For instance, techniques such as technology assessment and requirements analysis can help to identify use cases, constraints, and requirements that the new technology needs to fulfill.

Frameworks include, for example, design and usability frameworks such as the Health IT Usability Evaluation Model (Health-ITUEM) for evaluating mobile health technology [55]. This includes assessment of subjective properties of the technology from the perspective of users, which have been shown to be crucial to user adoption of technology, but that developers may not necessarily consider as a priority during the development process, including ease of use and perceived usefulness.

### Frameworks With a User Focus

While use is crucial for the successful development of AI-based technology, empirical work has shown that systems may be used in ways other than intended, which may in turn result in unanticipated threats to organizational functioning and patient safety [56]. For example, users may develop workarounds to compensate for usability issues of technologies, but these workarounds may compromise the intended performance of a system [57]. Frameworks that focus on the user of the technology can help to address these issues and facilitate the “optimization of technology use”. In doing so, they can help developers and implementers understand the various contexts of the use of AI-based technologies, as well as unintended consequences, and tailor systems to maximize benefits and minimize harms. For instance, a contextual analysis can help to gain a deep understanding of the various contexts in which a technology will be deployed. This includes examining cultural and social factors, as well as user behavior, user expectations, and existing systems or practices.

An example framework in this context is the Health Information Technology Evaluation Framework (HITREF), which includes an assessment of a technology’s impact on quality of care as well as an assessment of unintended consequences [58].

### Frameworks With an Organizational Focus

AI-based technologies are not adopted in a vacuum but must be integrated within organizational contexts. Previous work has shown that organizational strategies to implement health IT (HIT) and organizational cultures can have significant consequences for adoption and use [59]. For example, lack of integration with existing health information infrastructures can slow down system performance and impede practical use, and hence, impact adversely on safety and user experience [60]. Frameworks with an organizational focus can facilitate the development of “organizational strategies” to implement new technologies. In doing so, they can help AI system implementers integrate AI safely within existing organizational structures and processes. For instance, these can help to inform communication strategies, training programs, and support mechanisms to help users understand the benefits and risks of AI technologies and adapt to new roles and responsibilities.

An example of a framework with an organizational focus is the Safety Assurance Factors for Electronic Health Record Resilience (SAFER) guides, which help implementing organizations identify existing risks and facilitate the development of mitigation strategies to promote the effective integration of technologies within organizational processes [61].

## Discussion

A range of theory-informed evaluation frameworks for diverse kinds of HIT already exist [62]. Although not all of these may be relevant for AI-based applications, many aspects of existing frameworks are likely to apply. Exploring the transferability of these dimensions, therefore, needs to be a central component of work going forward [63].

Existing frameworks examine various aspects of technology design, implementation, adoption, and optimization. On the most basic level, they can be distinguished according to their focus, which then influences their application and context of use. A simplified overview of selected HIT evaluation frameworks and their potential application to AI is shown in Table 2. Frameworks with a technology focus can help to inform the conception and design of technologies through actively and iteratively involving end users, bridging the gap between technology development and application. This can, in turn, mitigate risks around nonadoption due to a lack of need or actionable system outputs. Frameworks with a user focus can help to ensure that systems are effectively embedded with adoption contexts and thereby mitigate the risk of systems not being used or not being used as intended. Finally, frameworks with an organizational focus can help to ensure that systems fit with existing organizational structures, and thereby help to ensure sustained use over time and across contexts.

We recommend that researchers, implementers, and strategic decision makers consider the use of existing theory-informed HIT evaluation frameworks before embarking on an AI-related initiative. This can help to mitigate emerging risks and maximize the chances of successful implementation, adoption, and scaling. To achieve this, existing and emerging guidelines for the evaluation of AI must promote the use of theory-informed evaluation frameworks.

Although many of the frameworks are well-known in the academic clinical informatics community, there is an urgent need to incorporate them into general AI design, implementation, and evaluation activities, as they can help to facilitate learning from experience and ensure building on the existing empirical evidence base. Unfortunately, this is currently not routinely done, perhaps reflecting disciplinary silos leading to lessons having to be learned the hard way. This, in turn, potentially compromises the safety, quality, and sustainability of applications. For example, although AI applications in radiology are now getting more established, the existing evidence base focuses on demonstrating effectiveness in proof-of-concept or specific clinical settings (the technology dimension in Table 2) [25]. Wider organizational and user factors are somewhat neglected, potentially threatening the wider sustainability and acceptability of such applications.

### Conclusions

We aimed to provide a conceptual overview of existing theory-informed frameworks that could usefully inform the development and implementation of AI-based technologies in health care, and we identified several frameworks with technological, user, and organizational foci. Future research could involve conducting a systematic review based on this pragmatic overview to synthesize existing evidence across evaluation frameworks, spanning the dimensions of technology, user, and organization.

Evaluation of AI-based systems needs to be based on theoretically informed empirical studies in contexts of implementation or use to ensure objectivity and rigor in establishing the benefits and thwarting risks. This will ensure that systems are based on relevant and transferable evidence and can be implemented safely and effectively. Theory-based HIT evaluation frameworks should be integrated into existing and emerging guidelines for the evaluation of AI [64-66]. The examples of frameworks provided could also help to stimulate the development of other related frameworks that can guide further evaluation efforts.

Drawing effectively on theory-based HIT evaluation frameworks will help to strengthen the evidence-based implementation of AI systems in health care and help to refine and tailor existing theoretical approaches to AI-based HIT. Learning from the wealth of existing HIT evaluation experience will help patients, professionals, and wider health care systems.

## Abbreviations

AI: artificial intelligence
Health-ITUEM: Health IT Usability Evaluation Model
HIT: health IT
HITREF: Health Information Technology Evaluation Framework
SAFER: Safety Assurance Factors for Electronic Health Record Resilience

## Data Availability

Data sharing is not applicable to this article as no data sets were generated or analyzed during this study.
